# Support vector machine and deep-learning object detection for localisation of hard exudates

**DOI:** 10.1038/s41598-021-95519-0

**Published:** 2021-08-06

**Authors:** Veronika Kurilová, Jozef Goga, Miloš Oravec, Jarmila Pavlovičová, Slavomír Kajan

**Affiliations:** Faculty of Electrical Engineering and Information Technology, Slovak University of Technology, Ilkovičova 3, 812 19 Bratislava, Slovakia

**Keywords:** Eye diseases, Computational biology and bioinformatics, Image processing, Machine learning

## Abstract

Hard exudates are one of the main clinical findings in the retinal images of patients with diabetic retinopathy. Detecting them early significantly impacts the treatment of underlying diseases; therefore, there is a need for automated systems with high reliability. We propose a novel method for identifying and localising hard exudates in retinal images. To achieve fast image pre-scanning, a support vector machine (SVM) classifier was combined with a faster region-based convolutional neural network (faster R-CNN) object detector for the localisation of exudates. Rapid pre-scanning filtered out exudate-free samples using a feature vector extracted from the pre-trained ResNet-50 network. Subsequently, the remaining samples were processed using a faster R-CNN detector for detailed analysis. When evaluating all the exudates as individual objects, the SVM classifier reduced the false positive rate by 29.7% and marginally increased the false negative rate by 16.2%. When evaluating all the images, we recorded a 50% reduction in the false positive rate, without any decrease in the number of false negatives. The interim results suggested that pre-scanning the samples using the SVM prior to implementing the deep-network object detector could simultaneously improve and speed up the current hard exudates detection method, especially when there is paucity of training data.

## Introduction

Hard exudates are composed of lipoprotein and lipid-filled macrophages that leak from impaired blood vessels. They can be found in the outer plexiform layer of the retina. In fundus photography, they appear as distinct wax lesions in the shape of clumps and rings. The presence of hard exudates is one of the main indicators of diabetic retinopathy^1^. The leakage of lipid-filled macrophages and fluid from the microaneurysms form retinal oedema. Hence, the presence of hard exudates is associated with retinal oedema^2^. The distribution of hard exudates and their relationship with the centre of the macula are crucial. The location of exudates within 500 μm of the centre of the macula is associated with the thickening of the retina. The existence of exudates in this area is part of the definition of clinically significant macular oedema^1^. The worsening of central vision associated with diabetic retinopathy is usually owing to diabetic macular oedema. Although the oedema remains largely undetectable under standard ophthalmoscopy or retinal fundus photography, identifying hard exudates is not difficult. In this work, we focused on creating an algorithm to automatically identify, locate, and segment hard exudates in retinal images to screen for diabetic retinopathy and the risk of macular oedema.

There are many methods for detecting or segmenting exudates. The first approach entails using image processing methods, sometimes in combination with machine learning methods, such as morphological operations^3^, fuzzy c-means clustering technique^4^, Kirsch’s edges^5^, stationary wavelets^5^, random forest algorithm^6^, bag of visual words^6^, and maximum margin SVM classifier^7^. Later works focused on deep-learning methods, mostly for image classification. Convolutional neural networks (CNN) can classify individual pixels as either exudates or non-exudates^8–10^, or classify image patches^11–13^. Deep neural networks can be based on various architectures pre-trained on different datasets^12–15^ (transfer learning method) or trained from scratch^16^. We decided to improve the effectiveness of the entire process by combining an SVM classifier and transfer learning methods^15,17^.

In our proposed method of object detection, we applied a CNN with a ResNet-50^18^ architecture trained on the ImageNet^19^ dataset for feature extraction. Based on the extracted features, the SVM classifier^20^, through rapid pre-scanning, divided the image samples into two categories, depending on the presence of hard exudates. The positive samples were then passed to a faster region-based convolutional neural network (faster R-CNN)^21^ object detector for detailed analysis and localisation of hard exudates. We evaluated the influence of SVM pre-scanning on the accuracy of hard exudate detection and localisation at two levels, the image and exudate level. We applied a five-fold cross-validation, with and without performing contrast augmentation on the training dataset and compared the results with those of previously published methods.

## Methods

**Figure 1:**
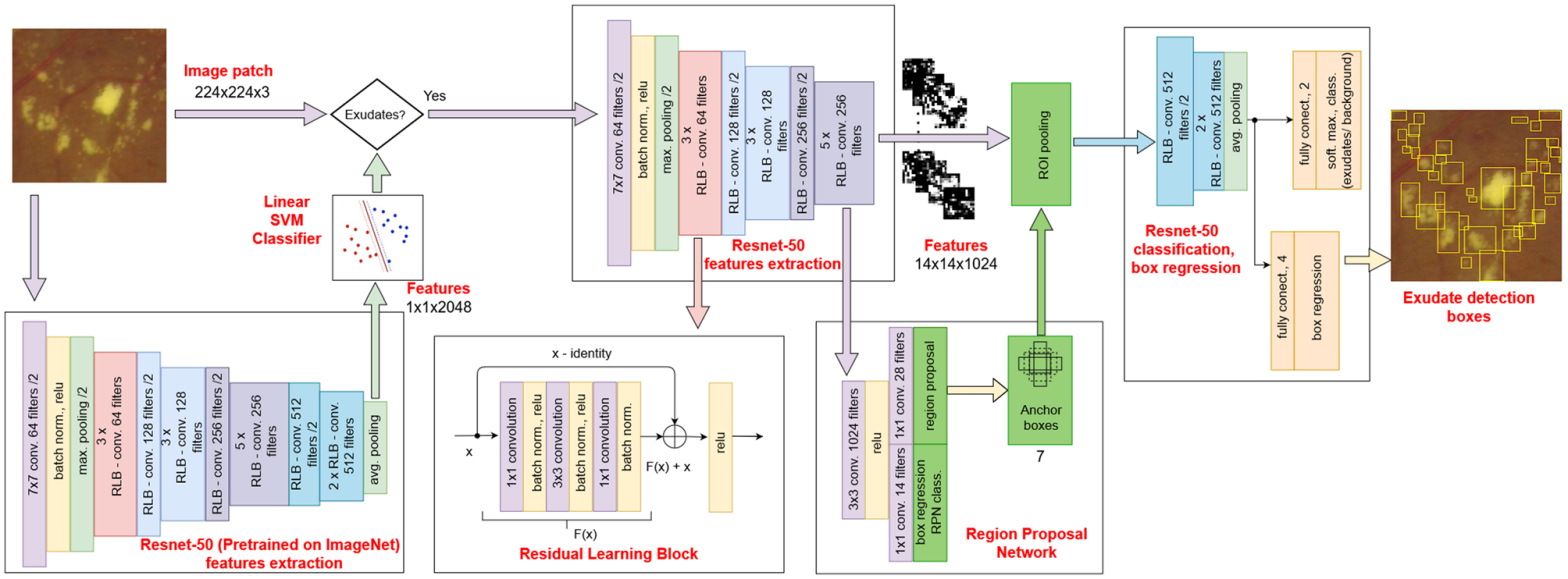
Proposed method for localisation of exudates on retinal images using a Resnet-50 object detector.

We propose a method for localising hard exudates on retinal images using a faster R-CNN^21^ object detector, based on the ResNet-50^18^ architecture in combination with an SVM classifier. To objectify our results, four variants of our method were developed: with and without the SVM classifier^20^ and with and without randomly adjusting the contrast of the dataset to augment it. For the variants with the SVM classifier, the input data consisting of image patches were pre-scanned using an SVM classifier. Only the patches that were classified as positive were further tested using the faster R-CNN object detector with a ResNet-50^18^ architecture trained on ImageNet^19^. A walkthrough of the schematic is shown in Fig. 1.

### Image acquisition

The number of available datasets of fundus images with manually marked exudates are limited. Therefore, for this study, it was important to find an appropriate database of fundus diabetic retinopathy images with precisely marked exudates. We chose the e-ophtha-EX^22^ database, a publicly available dataset with considerably heterogeneous images. The ground-truth data were the data manually marked with great precision pixel-by-pixel by two ophthalmologists. The training images, selected from this database, with different resolutions (i.e., 2544 × 1696, 2048 × 1360, and 1440 × 960 px), were not resized. Instead, because the inputs for ResNet must be 224 × 224 px, we added black columns and/or black rows to obtain resolutions that were divisible by 224 horizontally and vertically, to be able to split an image into patches. We decided not to change the size of the original images to avoid information loss. In addition, we increased data diversity because the training dataset contained objects of different sizes. We used all 47 images containing exudates and 35 images of healthy patients from the e-ophtha-EX^22^ dataset. The images containing exudates were randomly divided into five approximately equal folds (two folds of ten and three folds of nine images) for cross-validation. The images of the healthy patients were split similarly into five folds of seven images each. To facilitate comparison the results with similar work by other authors, we used the entire DiaretDB1^23^ and Messidor1^24^ datasets for external held-out testing. The DiaretDB1^23^ dataset consists of 89 images and ground-truth data with marked exudates in areas that are mostly larger than necessary. We divided this dataset into 38 and 51 images with and without exudates, respectively. In the DiaretDB1 dataset, an image was classified as containing exudates based on the judgement of 75% or more (at least, three out of four) of the experts. The Messidor1^24^ dataset consists of 1200 colour fundus images. The presence of hard exudates in the dataset has been used to grade the risk of macular oedema, as Risk 1 and Risk 2 groups. In this study, we combined both risk groups as a positive image class containing hard exudates. Because the exudates were not marked in this dataset, we evaluated whole images based on the occurrence of exudates.

### Preparing the training dataset for deep neural networks

**Figure 2:**
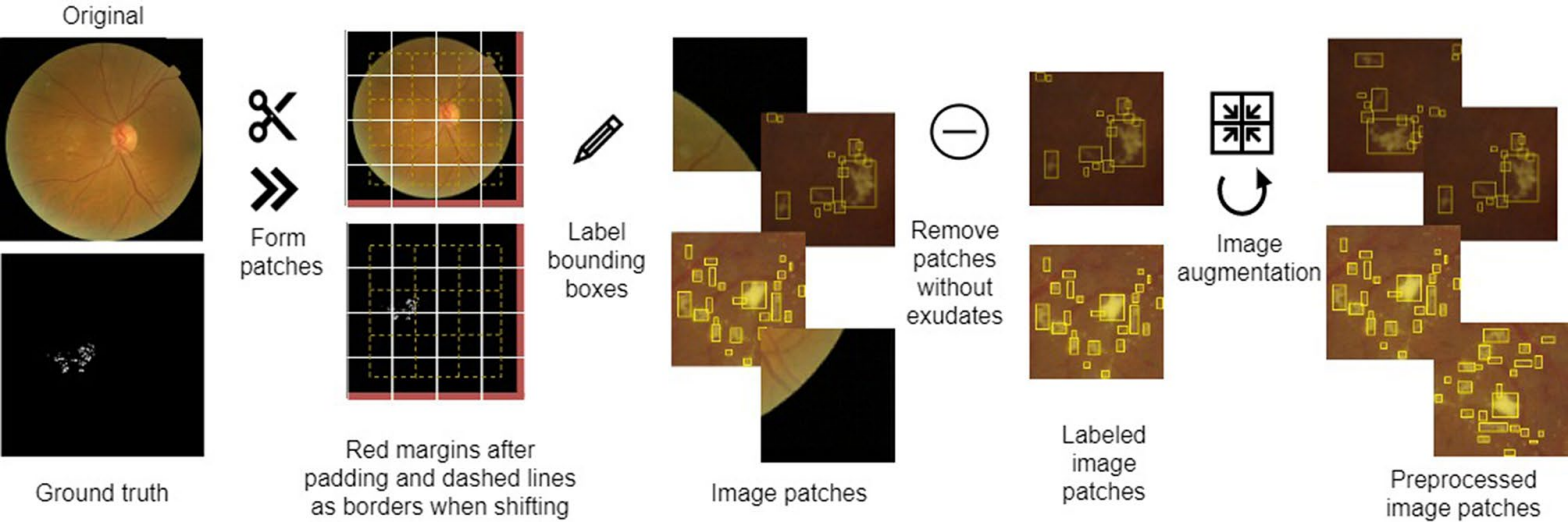
Creation of training data patches from retinal images and their ground-truth binary masks.

**Figure 3:**
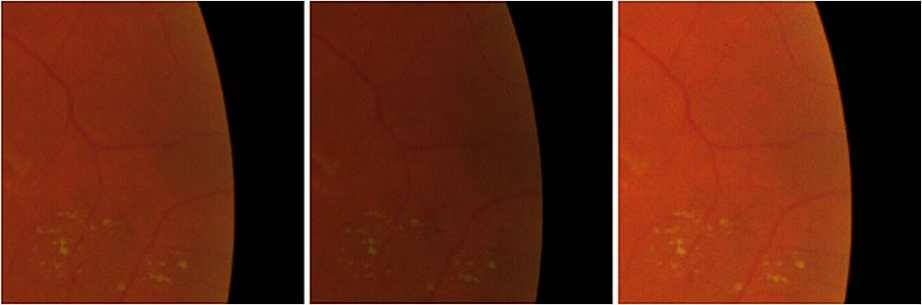
Example of random contrast adjustments made to augment the dataset for neural network training. The first image is of the original patch, the second and third images are of patches with random contrast and brightness adjustments in the range of 0–20% of the original image.

The process of creating training data patches for deep neural networks is illustrated in Fig. 2. The neural network training dataset consisted of only image patches with exudates. We set the rectangle bounds to the exudates in the ground-truth data to obtain rectangular coordinates in the form of neural network inputs. Considering the areas of the connected components in the binary ground-truth image, we automatically saved the coordinates of the upper left corner and the size of each bounding rectangle as one exudate. Each exudate was defined as an object that was not connected to the other marked objects. In most of the image patches, more than three exudates were observed. The training set consisted of 307 original image patches containing exudates (patches without exudates were not included in the training set), 882 shifted image patches, and 3567 mirrored image patches. To obtain a larger training set, we automatically created shifted image patches with a stride of 112 pixels, adopting different starting positions, namely, the vertical, horizontal, and both directions simultaneously. The borders of the shifted patches in both directions are indicated by dashed lines in the second column of Fig. 2. We also created mirrored image patches from the original patches by flipping each patch along the axes, similar to the shifted patches. To prevent the detectors from being over-trained in the deep network architecture^25,26^, we introduced random pixel-level transformations to achieve train-time augmentation. To extend the training set, we used random intensity and contrast variations within a range of 0% to 20%, while maintaining the original black background colour through thresholding. Examples of patches with adjusted contrasts are shown in Fig. 3. Random contrast adjustment augmentation of the dataset was performed on two variants of our method (with and without the SVM classifier).

### Preparing SVM dataset

Our SVM classifier was trained using all the images from the e-ophtha-EX dataset, in contrast to neural network training where only the image patches containing exudates were used. The images were chosen consistently according to cross-validation groups to train and evaluate five SVM classifiers, one for each validation fold. The images were divided into 224 × 224 px image patches using the same methodology as in the neural network training dataset. Each training fold contained twice as many non-exudate patches as exudate patches generated from the images containing exudates and healthy images by random selection. The inclusion of healthy retina patches was important because the appearance of a healthy retina ‘background’ (part of the retina without pathological findings) slightly differs from that of a retina ‘background’ with diabetic retinopathy. To identify and correctly classify patches with more black pixels, we added four pure black patches to the training folds. None of the other patches contained more than 33% of the black pixels and were randomly chosen.

### Linear SVM classifier

The SVM classifier is based on structural risk minimisation; it searches for a hyperplane in an N-dimensional space that can separate the data of different classes, that is, patches with and without exudates in our case. Support vectors are points lying on the hyperplane that support the optimal classification surface^20^. The classifier with a linear kernel function was trained using the features extracted from a Resnet-50 pre-trained on the ImageNet dataset. The SVM classifier and faster R-CNN detector were trained on the same exudate patches to avoid methodological errors. The extracted features from the last AvgPool layer with dimensions of 1 × 1 × 2048 that were obtained by passing training samples through the pre-trained ResNet-50 network were the inputs for the SVM classifier. The SVM classifier was trained for a binary classification task using a linear kernel with the cross-validation methodology similar to the faster R-CNN; training was performed with four folds and the classification accuracy was determined based on the percentage of correctly classified patches from the validation fold. Implementing this classifier as the first step before object detection served as the screening step through which the dataset was efficiently divided into patches with and without exudates. Only those with exudates were then referred to the object detector. We performed two experiments using the SVM classifier, with and without contrast adjustments.

### Faster R-CNN

There are three variants of R-CNNs, namely, R-CNN^27^, fast R-CNN^28^, and faster R-CNN^21^, which differ in terms of performance. The faster R-CNN is more efficient than previous networks. Instead of using edge boxes (an external algorithm used by the R-CNN and fast R-CNN), faster R-CNN features a region proposal network (RPN) for generating region proposals directly in the network. The architecture of a standard R-CNN consists of feature extraction layers and three classification layers. The last three classification layers of the R-CNN are replaced with new layers corresponding to the desired object classes, that is, dataset-specific features, a fully connected layer, softmax, and a classification layer. A box regression layer and a region-of-interest pooling layer are added to the fast R-CNN and the faster R-CNN contains an RPN^21^.

In this study, we used the faster R-CNN. The use of this network was inspired by some authors^29^ who recommended the use of faster R-CNN with the Inception V2 model when the goal is a highly accurate detection. First, the regions that might contain exudates (also called region proposals) must be determined. This was done using the region proposal function, which searches for subsets of images that may contain an object, for example, an exudate. The features from these region proposals were extracted and used for the classification of objects (in our case, whether the object is an exudate or background). The RPN uses anchor boxes for object detection. It is not necessary to scan the entire image using a sliding window to compute separate predictions for all positions. Anchor boxes are predefined bounding boxes of fixed size that are tiled across the image. Their dimensions were determined based on the sizes of the detected objects. The network predicts the probability of each anchor box. Using anchor boxes speeds up the detection process, enables multiple object detections, and detects overlapping objects and objects with different dimensions.

### Configuration of our faster R-CNN

The faster R-CNN in this study was trained using 4756 full-colour 224 × 224-px image patches prepared as described at the beginning of this section and their exudate coordinates. To determine the number of training epochs necessary, we used the first training set from cross-validation with a changed seed for a random number generator. We used the validation set to continuously evaluate the fitness function in the training process using an early stop if the error on the validation set worsened five times in a row. The validation criteria were met for ten epochs. Subsequently, the training was performed using a fixed number of epochs (ten) for both experiments (with and without contrast augmentation) without using an early stop while applying the five-fold cross-validation. The stochastic gradient descent with momentum was chosen as a solver with a mini-batch size of 2 and a fixed learning rate of 3 × 10^−4^. The negative and positive overlaps were set to [0 0.1] and [0.1 1], respectively. The training was performed using a graphics processing unit on an 8-core AMD FX8320 computer with 3.5 GHz, 16-GB RAM, Windows 10, 64-bit, GeForce GTX 1080, 8 GB hard disk, and Matlab R2019b.

### Evaluation of training and validation results

The results were evaluated at both the exudate and image levels, according to Zhang et al.^6^. In contrast to the results of Zhang et al.^6^, the results of our object-detection algorithm are rectangular boxes with their coordinates. To measure the accuracy of the pixels in the bounding boxes, we filled the rectangles from our neural network findings and compared them to filled rectangles created around exudates in the ground-truth data.

We evaluated the results of the four variants of our method: with and without the SVM classifier and with and without augmenting the neural network dataset through random contrast adjustments. The evaluation metrics were sensitivity, specificity, accuracy, and positive predictive value (PPV) using the standard formula:

1 $$\begin{array}{c}Sensitivity=\frac{True\, positives}{True \,positives+False\, negatives},\end{array}$$

2 $$\begin{array}{c}Specificity=\frac{True\, negatives}{True\, negatives+False \,positives},\end{array}$$

3 $$\begin{array}{c}Positive \,predictive\, value=\frac{True\, positives}{True \,positives+False\, positives},\end{array}$$

4 $$\begin{array}{c}Accuracy=\frac{True \,positives+True \,negatives}{True\, positives+True\, negatives+False\, positives+False\, negatives},\end{array}$$

To compare the results of our study with others, we evaluated the F1 score, which was computed using the following formula:

5 $$\begin{array}{c}F1 score=\frac{2\times sensitivity\times PPV}{sensitivity+PPV}.\end{array}$$

We computed the false positive and false negative rates to evaluate the contribution of the SVM classifier.

6 $$\begin{array}{c}False\, positive\, rate=\frac{False\, positives}{False\, positives+True\, negatives},\end{array}$$

7 $$\begin{array}{c}False\, negative \,rate=\frac{False \,negatives}{False \,negatives+True\, positives}.\end{array}$$

### Exudate-level evaluation

**Figure 4:**
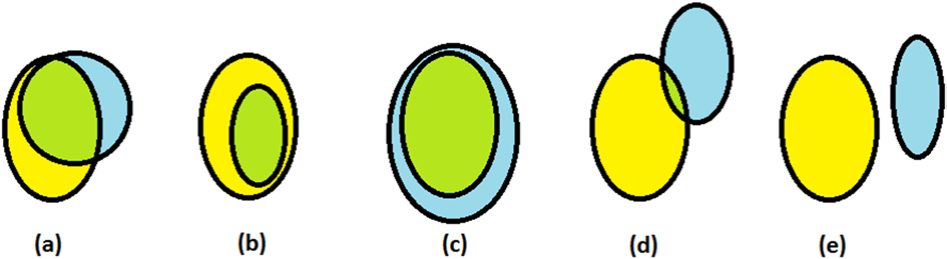
Schematic examples of the overlap evaluation (green colour) of candidate (blue colour) and ground truth (yellow colour). (**a–c**) were classified as found exudates. (**d,e**) were not classified as found exudates.

**Figure 5:**
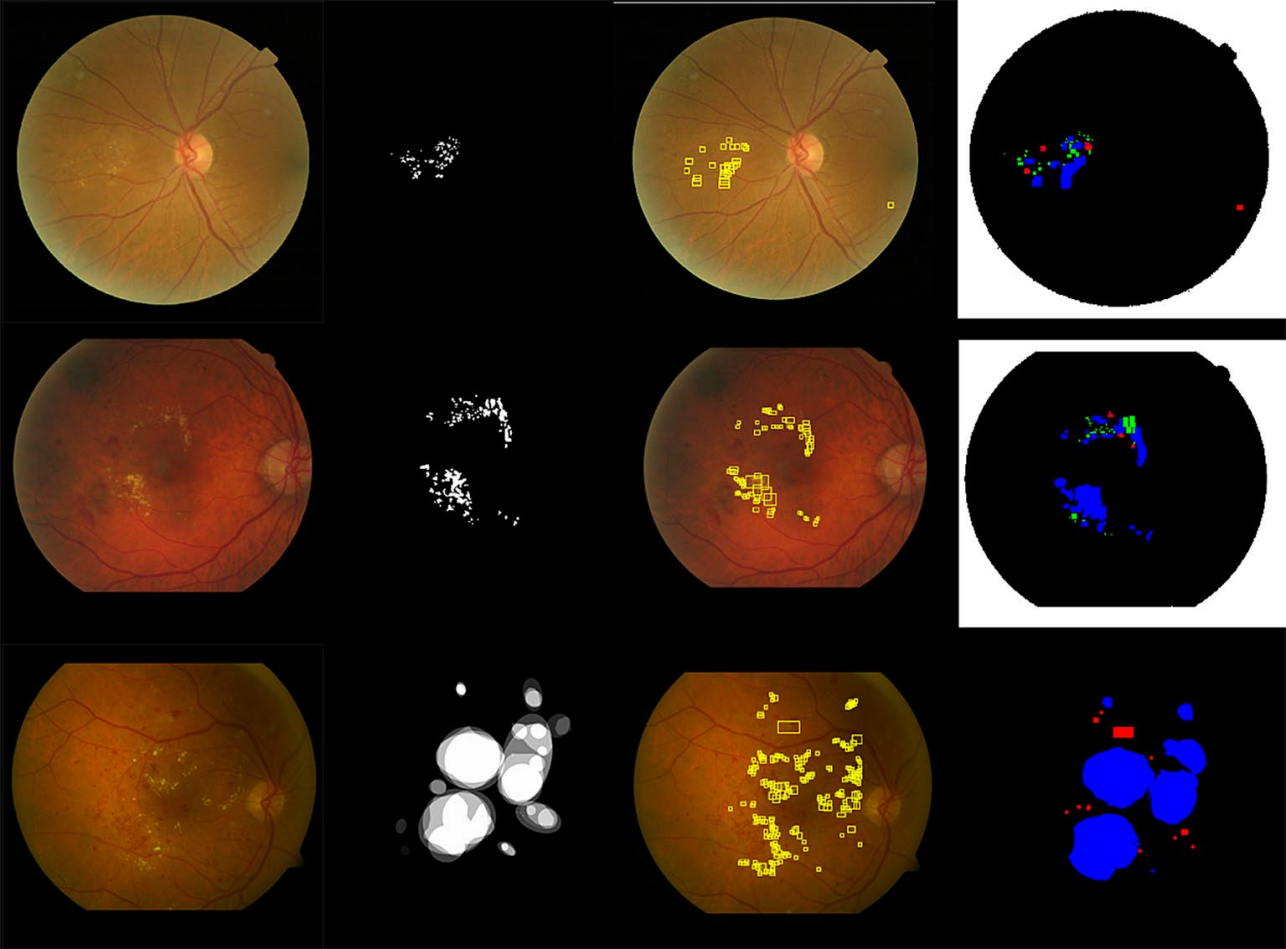
Results of the proposed method. First two rows are examples from the e-ophtha-EX dataset; last row is from the DiaretDB1 dataset. The first, second, third, and fourth columns show the original images, ground-truth data, object detection results, and evaluation map, respectively. The true positives, false negatives, and false positives are denoted in blue, green, and red, respectively.

Counting the correctly and incorrectly classified pixels proved ineffective^6^. Therefore, we evaluated the results by measuring the overlap between the ground-truth data and the objects detected by our neural network. The main goal was to correctly classify the exudate based on the overlap between the detected object and the ground-truth data. The true positives were the pixels of exudates with an overlap of more than 20% between the detected object size and the ground-truth exudate size. The false positives were the pixels of exudates with an overlap of 20% or less between the detected object size and the ground-truth exudate size. The false negatives were pixels with an overlap of 20% or less between the ground-truth exudate size and ground-truth data with no overlap with any of the detected objects. Finally, all the remaining pixels were marked as true negatives. Schematic examples of the evaluation of the overlap between the candidate data and ground-truth data are shown in Fig. 4. This overlap-based evaluation approach at the exudate level was used when the detector was tested on the e-ophtha-EX database because it contains precise ground-truth data (each exudate is marked pixel-by-pixel). A slightly different approach was taken to evaluating the performance of the faster R-CNN detector in the DiaretDB1 dataset because of large areas around exudates in the ground-truth data. The large areas marking the exudates in DiaretDB1 are shown in Fig. 5 (second column of the last row). An object was counted as an exudate when there was any overlap with these large ground-truth data.

### Image-level evaluation

The image-level results were based on the values of the image patch probabilities. We evaluated the likelihood of an image containing an exudate based on the maximum patch probability score. The image was considered positive if at least one patch probability was equal to or higher than 0.9. This approach may be more important from a clinical point of view^16^.

## Results

### SVM classifier accuracy

We evaluated the ability of the SVM classifier to correctly classify image patches based on the exudate occurrence and recorded a cross-validation accuracy of 84.7%. This value represented the average classification accuracy of all the validation folds. The maximum classification accuracy was 92.2% for one validation fold. The contribution of the SVM pre-scanning is presented in the following sections.

### Exudate-level results

The results for all the four variants of the proposed method at the exudate-level, computed with five-fold cross-validation using the e-ophtha-EX dataset on images with exudates are shown in Table 1. The best sensitivity and lowest false negative rate were achieved without the SVM classifier. In contrast, the highest F1 score and lowest false positive rate were achieved with the SVM classifier. The variants of the proposed method when the neural network training dataset was not augmented through contrast adjustment exhibited better performance. In conclusion, the SVM classifier increased the F1 score by reducing the false positive rate by 29.7% and 23.4% with and without contrast adjustment, respectively. The SVM classifier also caused a slight decrease in the sensitivity owing to a slight increase in the false negative rate by 18% and 16.2% for the variants of the proposed method with and without contrast adjustment, respectively. This was notable for both variants of the proposed method. The SVM classifier also accelerated the testing process of the images. The colour depiction of positive and negative pixels in the validation datasets is shown in the first two rows of Fig. 5.

**Table 1:** Sensitivity, specificity, accuracy, F1 score, PPV, false positive rate (FPR) and false negative rate (FNR) at the exudate and image level of the proposed method are shown in the table.

Performance metric	Sensitivity	Specificity	Accuracy	F1 score	PPV	FPR	FNR
**Exudate-level**							
Faster RCNN	0.8987 ± 0.0752	0.9953 ± 0.0019	0.9947 ± 0.0017	0.6781 ± 0.0378	0.5984 ± 0.0593	0.0047 ± 0.0019	0.1013 ± 0.0752
Faster RCNN + SVM	0.8805 ± 0.0672	0.9964 ± 0.0012	0.9957 ± 0.0012	0.69262 ± 0.0462	0.6236 ± 0.06208	0.0036 ± 0.0012	0.1195 ± 0.0672
Faster RCNN + CA	0.8575 ± 0.0492	0.9963 ± 0.0016	0.9956 ± 0.0014	0.6577 ± 0.0103	0.6432 ± 0.1023	0.0037 ± 0.0016	0.1425 ± 0.0492
Faster RCNN + CA + SVM	0.8344 ± 0.0442	0.9974 ± 0.0009	0.9966 ± 0.0009	0.7069 ± 0.0753	0.6626 ± 0.0982	0.0026 ± 0.0009	0.1656 ± 0.0442
**Image-level**							
Faster RCNN	1	0.6286 ± 0.0782	0.8412 ± 0.0346	0.8786 ± 0.0244	0.7841 ± 0.0392	0.3714 ± 0.07825	0
Faster RCNN + SVM	1	0.7143 ± 0.1010	0.8779 ± 0.0418	0.9044 ± 0.0299	0.8266 ± 0.0508	0.2857 ± 0.1010	0
Faster RCNN + CA	0.9578 ± 0.0579	0.4857 ± 0.2595	0.7566 ± 0.1129	0.8222 ± 0.0713	0.7254 ± 0.1000	0.5143 ± 0.2595	0.0422 ± 0.0579
Faster RCNN + CA + SVM	0.9578 ± 0.0579	0.7429 ± 0.1863	0.8669 ± 0.0768	0.8940 ± 0.0550	0.8429 ± 0.0839	0.2571 ± 0.1863	0.0422 ± 0.0579

The first column shows the four variants of our method: faster R-CNN with or without contrast adjustment augmentation (CA) of the dataset and with or without SVM classifier pre-scanning. The values are represented as µ ± σ; µ represents the mean and σ represents the standard deviation.

### Image-level results

**Figure 6:**
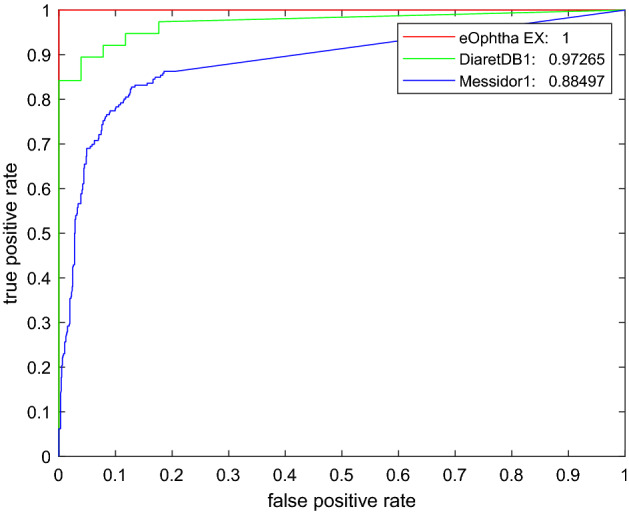
AUC-ROC curve at the image-level of our method on the e-ophtha-EX, DiaretDB1, and Messidor1 datasets.

The results for all the four variants of the proposed method at the image-level are presented in Table 1. The SVM classifier improved the F1 score, with a reduction in the false positive rate by 50.00% and 23.07% with and without the contrast adjustment augmentation of the dataset, respectively, without any decrease in sensitivity or rise in false negatives. In the evaluation, the results of the SVM classifier at the image-level were markedly better, compared with the exudate level. This was more evident for the variant using the faster R-CNN with the dataset augmented through contrast adjustment. We also computed the area under the receiver operating curve (AUC) using the results of the faster R-CNN with the contrast adjustment variant of the proposed method, as shown in Fig. 6.

### Comparison with other methods and results of the held-out test

Our results on the e-ophtha-EX dataset were compared with those of the other methods in Table 2. The article authors are listed in the first column; the results at the exudate and image levels are summarised in the second column and in the next two columns, respectively. Examples of our results are shown in Fig. 5. We achieved an F1 score of 0.8367 at the exudate level using the best validation dataset with contrast adjustment augmentation, outperforming all the aforementioned methods. For the held-out test, the F1 scores for DiaretDB1 were 0.8898 and 0.881 for the exudate- and image-level evaluations, respectively. In addition, the AUC at the image level was 0.9727 for the DiaretDB1. Our results for the DiaretDB1 held-out test outperformed all the other methods at both the exudate and image levels. Only image-level evaluation was performed on the Messidor1 database, resulting in an AUC of 0.885. The AUC-ROC curves for all the three datasets are shown in Fig. 6. The AUC-ROC curve for the e-ophtha-EX dataset was plotted from the results of the best-performing validation dataset for the neural network variant of the proposed method with contrast adjustment augmentation using the SVM classifier.

**Table 2:** Results of the proposed method, compared to other methods.

Author	Exudate-level: F1	Image-level: F1	Image-level: AUC
**e-ophtha-EX dataset**			
Giancardo, applied by Zhang^6^	–	–	0.87
Zhang^6^	0.732	–	0.95
Abbasi-Sureshjani^16^	0.832	0.967	0.994
Our method	**0.8367**	**0.9474**	**1**
**DiaretDB1 dataset**			
Giancardo^30^	–	–	0.93
Zhang^6^	–	–	0.95
Abbasi-Sureshjani^16^	0.819	0.880	0.965
Our method	**0.8898**	**0.881**	**0.9727**
**Messidor dataset**			
Giancardo^30^	–	–	0.88–0.89
Our method	–	–	**0.885**

Results of the proposed method are highlighted in bold.

## Discussion

We propose a method for detecting hard exudates using a deep-learning object detector. Four variants of our method were evaluated at the exudate and image levels: with and without SVM pre-scanning and with and without random contrast adjustments of the dataset evaluated through fivefold cross-validation. The combination of the faster R-CNN detector and the SVM classifier with contrast adjustment augmentation achieved the best F1 score of 0.8367 at the exudate-level. The F1 score improved, with a slight decrease in sensitivity. None of the other methods achieved an F1 score above 0.832^16^.

When evaluating the held-out test, we applied a different evaluation approach from the one used by Abbasi-Sureshjani et al.^16^. In contrast to their method, where the exudates in the ground-truth data were considered as exudates when marked by more than 75% of experts, we considered exudates if they were annotated by 75% of experts or more. This means that we considered account markings from at least three out of four experts, rather than all four experts. This evaluation approach yielded 30 and 38 images with exudates in the work of Abbasi-Sureshjani^16^ and in this study, respectively. With this approach, smaller and less notable exudates (marked on the ground-truth data) were included among the diagnosed objects. Despite this more stringent specification, we obtained better results. Our results on the Messidor1 dataset were similar to those obtained by Giancardo^30^ when training on different datasets; however, the dataset only contains the grading of the risk of macular oedema. It does not contain marked exudates.

The SVM classifier was beneficial for reducing false positives in both evaluation approaches, with and without contrast adjustments. The SVM classifier based on the extracted features from the pre-trained ResNet-50 network was effective at reducing false positives arising from various factors, such as images of poor quality, artefacts, nerve fibre reflections, lesions after laser therapy, and pigment epithelial changes, although it achieved only 84.7% average accuracy. These false positives should be reduced intensively when training more or larger datasets with exudates marked pixel-by-pixel, as in the e-ophtha-EX^22^ dataset. Mild reduction in sensitivity and rise in false negatives at the exudate-level evaluation because of the SVM classifier could be acceptable because of its huge improvement potential for the image-level evaluation, which tends to be clinically more important and noticeable across all the variants of the proposed method. The proposed method outperformed other supervised exudate detection or segmentation methods evaluated by Zhang^6^.

A limitation of this study was that the chosen object detection algorithm was allowed to train on image patches with exudates only and no patches of healthy fundus images were included. Although these image patches contained exudates and background (non-exudate part of the image patch), the appearance of this background slightly differed from those found without exudate patches. There were also significant differences in the background appearance between diabetic retinopathy and healthy fundus images. To obtain better results and cover the entire colour spectrum of retinal images, we added more image patches by randomly varying the contrast for data augmentation. Remarkably, these variants of our method were less sensitive, and we noted a decrease in the FPR at the exudate-level. Overall, these variants led to less detection, compared to variants that did not use contrast-change dataset augmentation.

It is more complicated to evaluate the results of a deep-learning object detector, than to evaluate the results obtained by semantic segmentation neural network, according to Zhang^6^. In our case, we had to create bounding boxes around the ground-truth exudates and compare the area in these bounding boxes with filled bounding boxes obtained through a neural network object detector. In contrast to semantic segmentation, this method detected every exudate as a separate object bordered by a bounding box and the coordinates of every exudate were direct outputs of the object detector.

Analysing images with a deep-learning object detector is time-consuming. The analysis of one retinal image in our implementation environment lasted from a few seconds to two minutes, depending on the severity of the diabetic retinopathy findings. The duration of this process was reduced by the SVM pre-scanning (scanning of the image before object detection). Scanning the patch using an SVM classifier was fast and allowed the exclusion of non-exudate image patches from the detection process. This became evident when testing on a computer with lower computational capacity.

Although our method is only an algorithm and not the entire solution, it can be incorporated into several clinical applications. It can also be implemented as a part of the diabetic retinopathy screening in telemedicine. The use of deep-learning methods to screen for diabetic retinopathy in telemedicine has been found to be as effective as when retinal images are examined by a human expert^31^. Deep-learning methods have made considerable progress in the segmentation of retinal vasculature. However, detecting retinal pathologies using these techniques remains underexplored^32^. The proposed deep-learning hard exudate detection with SVM pre-scanning is a method of retinal pathology detection that can enrich current telemedicine solutions with precise quantity, size, and localisation of hard exudates. Determining the position of hard exudates can be a part of the diabetic macular oedema prediction algorithm. However, screening for diabetic macular oedema alone is currently not advised^33^. The essential parts of diabetic macular oedema examination and follow-up are fluoroangiography and ocular computer tomography examinations^34^. However, these examinations are not affordable for all eye care practitioners. In addition to a standard slit lamp examination, in the fundoscopic and panfundoscopic examinations, fundus cameras are an inexpensive solution for common eye care practitioners to follow-up and archive the findings of diabetic retinopathy patients. The proposed hard exudate detection and localisation system can be a useful part of the solution that estimates, archives, and follows up on the quantity and localisation of hard exudates in diabetic retinopathy patients of common eye care practitioners. Assessing every exudate as an object during fundus photography can be the first step towards analysing them quantitatively and spatially. The risk of developing diabetic macular oedema can be computed based on these data.

Deep-learning exudate detection in fundus images have potential applications also outside ophthalmology. Fundus image analysis based on deep-learning techniques can predict cardiovascular risk factors^35^ or anaemia^36^. The quantity of hard exudates is related to serum lipid levels, and the risk of central localisation of hard exudates in patients with higher serum triglyceride levels is higher^37^. A higher prevalence of diabetic macular oedema has also been associated with higher serum cholesterol levels^38^. Therefore, predicting higher lipid levels based on the quantity and localisation of hard exudates can be an interesting future research direction.
